# Oil-impregnated densified wood veneer with high electrical insulation enabled by nanosized oil channels

**DOI:** 10.1126/sciadv.aed5744

**Published:** 2026-06-10

**Authors:** Meiling Wu, Yue Xu, Qian Zhang, Alexandra Brozena, Yimin Mao, Yu Liu, Lin Xu, Soori Tejaswi, Yugang Zhang, Xinpeng Zhao, Kent Congxi Zhang, Kishore Ramakrishnan, Oliver Wang, Keith Nelson, Vaibhav Bahadur, J. Y. Zhu, Robert E. Hebner, Liangbing Hu

**Affiliations:** ^1^ Department of Materials Science and Engineering, University of Maryland, College Park, MD 20742, USA.; ^2^ Center for Electromechanics, University of Texas at Austin, Austin, TX 78758, USA.; ^3^ Department of Electrical and Computer Engineering, Department of Materials Science, Center for Materials Innovation, Yale University, New Haven, CT 06511, USA.; ^4^ Walker Department of Mechanical Engineering, University of Texas at Austin, Austin, TX 78712, USA.; ^5^ Center for Functional Nanomaterials, Brookhaven National Laboratories, Upton, NY 11973, USA.; ^6^ InventWood Company, Frederick, MD 21703, USA.; ^7^ Rensselaer Polytechnic Institute, Troy, NY 12180, USA.; ^8^ US Department of Agriculture (USDA) Forest Products Laboratory, Madison, WI 53726, USA.

## Abstract

Growing energy demands and renewable integration are stressing the aging power grid infrastructure. Lignocellulosic oil-impregnated paper is widely used in power transformers but suffers from critical limitations, such as low dielectric strength, mechanical strength, and thermal conductivity, causing premature transformer failures. Here, we demonstrate a superior electrically insulating oil-impregnated paper design using the naturally anisotropic structure of densified wood veneer to achieve nanosized channels of oil that efficiently disrupt electrical breakdown pathways. The developed oil-impregnated densified wood (ODW) creates aligned cellulose fibers with 166 ± 87–nanometer oil nanochannels, achieving record dielectric strength of 105 kilovolts per millimeter. The structure also delivers a mechanical strength of up to 384 megapascals and a thermal conductivity of 0.33 watts per meter per kelvin, enabling enhanced longevity upon thermal aging tests. The ODW could replace conventional transformer insulation to enhance power transformer performance and improve lifetime. Moreover, its anisotropic oil-filled nanochannel design offers a general strategy for hybrid dielectrics in medium- and high-voltage applications.

## Teaser

An oil-impregnated densified wood with nanosized oil channels achieves record dielectric strength to extend transformer lifespan.

## INTRODUCTION

**Fig. 1.**
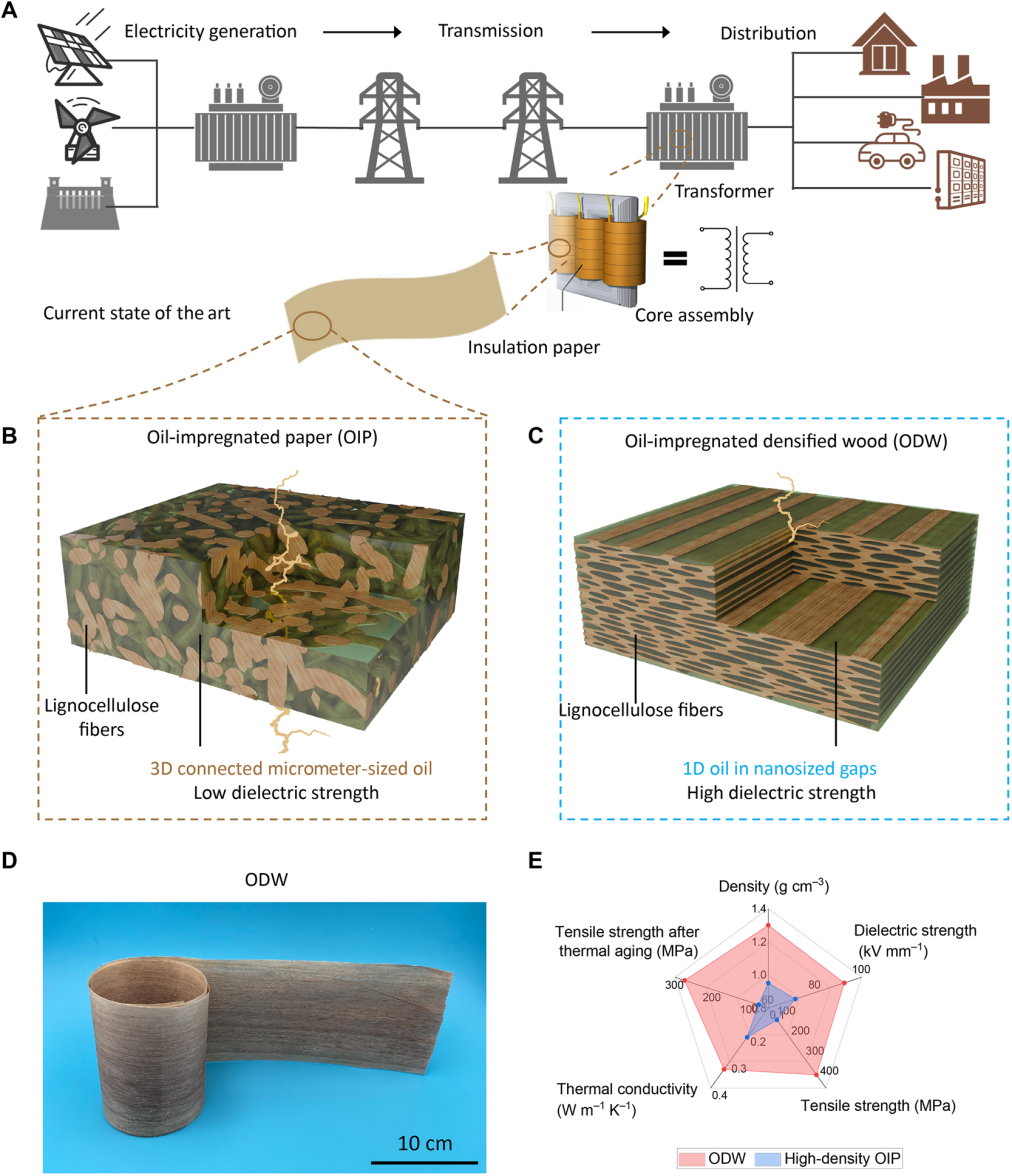
Fabrication and performance of the ODW for power transformer insulation. (**A**) Schematic illustration of the power grid, in which the transformer plays an important role in the transmission and distribution of electric power. The insulation paper in the core assembly provides electrical insulation between the core and windings, as well as the required insulation between the high- and low-voltage windings. Images used courtesy of ANSYS, Inc. (**B** and **C**) Schematics of the oil and cellulose distribution in the (B) conventional OIP and (C) proposed ODW, as well as exemplary electrical breakdown paths, which are indicated by the yellow lines. The conventional OIP structure features interconnected micrometer-sized oil pockets within the randomly distributed lignocellulosic fibers. In contrast, the ODW structure displays isolated and nanosized channels of oil between highly aligned and densified cellulose fibers. (**D**) Photo of a roll of ODW with a thickness of ~70 μm. (**E**) Performance comparison of ODW and high-density OIP with densities of 1.3 and 0.95 g cm^−3^_,_ respectively.

Increasing reliance on intermittent sources of renewable energy combined with growing energy demands (such as data centers and electric vehicle charging) has created the urgent need to build better electric grid infrastructure with high performance, enhanced reliability, and longer lifetime (*1*–*4*). Notably, the failure of power transformers can easily lead to wide power outages, with insulation failure being the largest cause and stemming from electrical, thermal, and mechanical stress (*5*–*7*). Plant-based lignocellulosic insulation paper dates back to the 1890s but is still widely used in power transmission and distribution systems today due to its low cost, good dielectric strength (~72 kV mm^−1^), and reasonable mechanical strength (~100 MPa) (Fig. 1A) (*8*, *9*). To improve the paper’s dielectric strength and the dissipation of heat generated during the operation of power transformers, it is necessary to exclude air from the paper structure by saturating the material in mineral oil or natural ester oil. However, one of the primary problems with conventional oil-impregnated insulation paper (OIP) is its structure, in which the oil is distributed within the networks of wood fibers, forming a three-dimensional (3D) interconnected network of micrometer-sized oil pockets (Fig. 1B). Because insulating oils have a lower dielectric breakdown strength than wood fibers (15 to 40 kV mm^−1^ versus 70 to 130 kV mm^−1^) (*10*, *11*), electrical breakdown occurs within these oil pocket networks, which negatively affects the dielectric strength. High-density OIP can increase the dielectric strength to around 72 kV mm^−1^ by decreasing the fraction of oil with low dielectric strength; however, improvement is limited. In practice, although lignin and hemicelluloses are known to negatively affect transformer insulation by accelerating aging, small amounts of these components are intentionally retained in conventional OIPs to achieve a balance between paper mechanical strength and dielectric performance (*12*, *13*). However, conventional OIPs are predominantly manufactured from wood fibers by removing a portion of lignin and hemicelluloses through the time- and energy-intensive kraft chemical pulping process. This pulping process inevitably degrades the cellulosic fibers, resulting in a decrease in cellulose degree of polymerization (DP) (*14*). Consequently, the conventional OIP is susceptible to long-term degradation under transformer operational conditions. In addition, conventional OIPs have a low thermal conductivity to result in ineffective heat transfer within the transformers (*15*) and a high transformer operation temperature. Fast material degradation under high temperatures, along with limited initial cellulose DP, results in gradually brittle insulation paper and eventual electrical breakdown (*6*, *16*, *17*). Various strategies for material innovation, such as incorporating nanofillers with high dielectric strength into the paper (*18*–*20*) and using three-layer cellulose-based paper (*7*), have been studied, but all failed to collectively improve mechanical and dielectric strength (which remains below 70 kV mm^−1^), as well as thermal conductivity, in addition to scalability challenges. The observed marginal improvement in dielectric strength may result from the 3D distribution of the insulating oil in the oilpaper composite, in which the electrical breakdown path can propagate along its abundant 3D interconnected oil network with limited cellulose involvement.

Here, we propose a paper insulation structure based on oil-impregnated densified wood (ODW) veneer that displays high dielectric strength, high mechanical strength, and high thermal conductivity, resulting in a long life under thermal degradation (Fig. 1, C to E). On the basis of rotary cut wood veneer featuring high alignment of the lignocellulosic fibers in the material’s plane direction, the insulating oil is confined between the partially delignified fibers, which better isolate the oil interconnections along the paper thickness direction. After densification, the insulating oil channels are further reduced to nanometer scale, with an average size of 166 ± 87 nm. The resulting laminated ODW structure exhibits a great dielectric strength of up to 105 kV mm^−1^, mostly due to the disruption of electrical breakdown paths between the isolated 1D nanoscale oil channels that run parallel through the ODW (Fig. 1C), as suggested by finite element simulations. Moreover, the increase in the breakdown strength is also contributed by the decreasing oil volume fraction, as oil has a lower dielectric strength than cellulose (15 to 40 kV mm^−1^ versus 70 to 130 kV mm^−1^, respectively). Furthermore, the high alignment and dense structure of the ODW fibers enable a high mechanical strength of up to 384 MPa and a thermal conductivity of 0.33 W m^−1^ K^−1^, increases of 3.5 and 1.57 times, respectively, compared with high-density OIP (Fig. 1E). These features provide the ODW a long life against thermal aging, i.e., retaining a mechanical strength of 274 MPa after 6 weeks at 150°C. The processing of ODW is also compatible with roll-to-roll manufacturing, enabling good scalability. As a proof of concept, we applied the ODW as insulation for a planar transformer and observed a temperature reduction of ~10°C compared with the conventional plastic insulation, demonstrating the higher thermal conductivity and the ODW’s applicability in a real planar transformer. The proposed ODW provides a competitive candidate for the next-generation oil-impregnated insulation material with comprehensive properties to support a reliable and resilient power grid. Moreover, the densified wood skeleton with aligned fibers and channels could also be used to improve the design of dry-type transformers impregnated with epoxy. Overall, we believe that this concept of using an anisotropic material to create 1D nanosized channels of air-excluding medium could serve as a promising design approach for hybrid electrical insulation materials, improving performance and reliability.

## RESULTS

### Fabrication and characterization of ODW

**Fig. 2.**
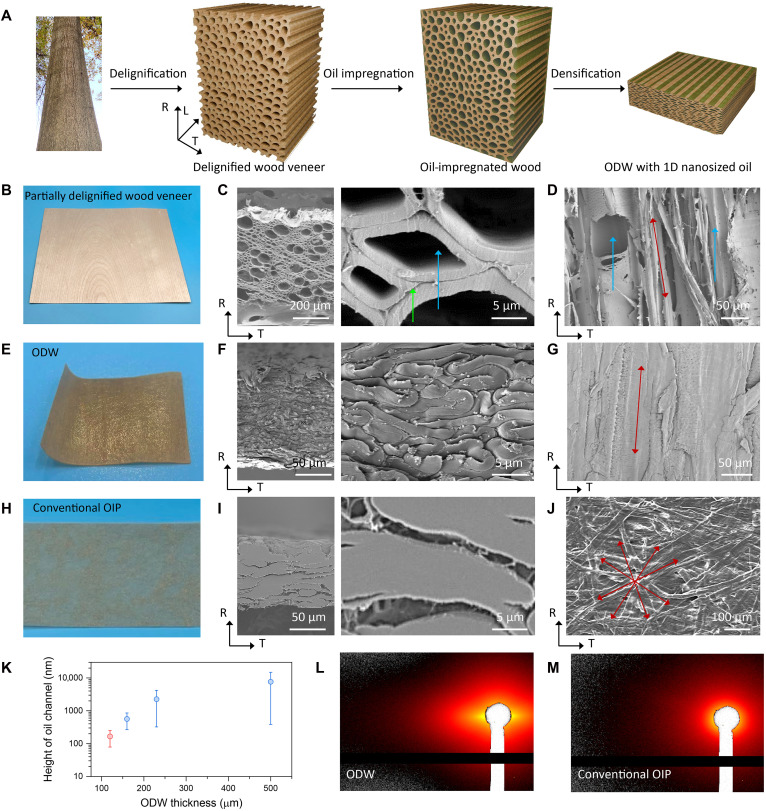
Morphology and structural characterization of the ODW. (**A**) Schematic of the fabrication of the ODW. Step 1 is a chemical treatment to partially remove lignin and hemicelluloses from the wood veneer. Step 2 is oil impregnation into the pores of the wood. Step 3 is mechanical pressing, which results in a densified paper containing oil. R, L, and T stand for Radial, Longitudinal, and Tangential, respectively, which are the three principal directions of wood regarding cutting or grain orientation. (**B**, **E**, and **H**) Digital photograph, (**C**, **F**, and **I**) cross-sectional, and (**D**, **G**, and **J**) top-down view SEM images of [(B) to (D)] partially delignified wood veneer, [(E) to (G)] ODW, and [(H) to (J)] conventional OIP. Note that the oil in ODW and OIP are removed by hexane for SEM characterization. The micrometer-sized lumina channels (indicated by blue arrows) and gaps between lumina channels (indicated by a green arrow) shown in the partially delignified wood veneer are fully compressed in the ODW, while conventional OIP features 3D interconnected space that can be filled with oil. (**K**) Height of the oil channels in the ODW samples with different thicknesses after densification. Data in (K) are presented as mean values ± SD based on at least 50 measurements. The red double arrows indicated fiber orientation in the wood veneer samples and OIP. (**L** and **M**) SAXS pattern of the (L) 120-μm-thick ODW and (M) conventional high-density OIP.

To fabricate the ODW (Fig. 2A), we take advantage of the naturally anisotropic structure of wood, which has an hierarchical pore structure including abundantly aligned macro-, micro-, and nanoscale channels constrained by the cell walls and constituent cellulosic fibers, fibrils, microfibrils, etc. (*21*). These natural pores can potentially be used for oil impregnation. However, wood also features lignin and hemicelluloses, which bind the cellulose fibers together and create low interfiber/fibril porosity that hinders oil infiltration, in addition to limiting the wood’s flexibility and processability (*22*). Therefore, to simultaneously increase wood porosity while minimizing degradation of cellulose DP, we applied a mild alkaline delignification by boiling natural wood veneer (basswood, 500 μm in thickness) in 5 vol % NaOH for 2 hours. This process partially removes lignin and hemicelluloses, resulting in a loosened wood structure with enlarged lumina channels from 1 to 50 μm and gaps between lumina channels from ~10 nm to 2 μm (Fig. 2, B to D) running along the wood fiber direction. In addition, the delignification treatment exposed the aligned cellulose comprising the cellulose fibers/fibrils (fig. S1A), thereby resulting in a less rigid and more porous wood structure (*22*). We then filled the voids in the delignified wood veneer with insulating oil (natural ester oil) under vacuum (see Materials and Methods for details). To prevent the incursion of air and moisture into the wood, which would reduce the dielectric strength, we pretreated the insulating oil under vacuum and 105°C to obtain an air/moisture-free insulating oil. After oil impregnation, we pressed the sample at a pressure of 7 MPa, thereby reducing the wood thickness to ~120 μm. This process removed excess oil and shrank the micrometer-sized lumina channels and the gaps between them to form a densely packed ODW laminated structure (Fig. 2, E to G, and fig. S1C). The remaining lignin likely helps maintain fiber cohesion and contributes to this densified, laminated structure during cold pressing. Consequently, any residual oil becomes confined into the shrunken lumina space and pores within the cellulose fibers. The predominantly open nature of the lumen voids and intercellular gaps facilitates completely displacing air by insulating oil during the described process, thereby mitigating the formation of air pockets. For comparison, we purchased commercial high-density OIP (i.e., “conventional OIP”) with a density of 0.95 g cm^−3^ and a thickness of 135 μm, which is also impregnated with the same insulating oil using a procedure similar to that used for ODW. Notably, compared with the ODW, the conventional OIP features less alignment of the cellulose fibers (Fig. 2, H to J, and fig. S1B) with a 3D interconnected distribution of oil within the voids.

By controlling the compression pressure from 1 to 7 MPa, we obtained ODW samples with different thicknesses (500 to 120 μm) after densification, which allowed us to control the height of the 1D channels that run through the material (fig. S2). These 1D channels were formed by the shrunken lumina and the gaps between two neighboring cells created by delignification (as indicated by the blue arrow and green arrow, respectively, in Fig. 2C). To determine the channel heights, we used scanning electron microscopy (SEM) to measure the gaps within the shrunken cells and between neighboring cell walls. As shown in Fig. 2K, the channel height is 167 ± 87 nm (7-MPa compression, corresponding to the 120-μm-thick sample), compared with an average original lumen height of 7.6 ± 7.3 μm (without densification). This suggests that nanosized 1D channels are successfully fabricated in the ODW. To determine the oil distribution at a macroscopic scale, we applied the small-angle x-ray scattering (SAXS) to investigate the orientation of the cellulose fibers of the ODW and OIP samples. The rhombus-shaped scattering pattern observed in the SAXS analysis of the ODW (Fig. 2L) indicates high alignment of cellulose nanofibers, whereas the conventional OIP shows a circular shape (Fig. 2M), which suggests a less-aligned distribution of the cellulose fibers. Thus, the insulating oil impregnated into the voids of ODW may also follow a high alignment behavior, suggesting the formation of 1D insulating oil channels at the macroscopic scale.

To further investigate where the insulating oil is located in the ODW structure, we performed 1D SAXS analysis to monitor the shrunken lumen voids and interfiber gaps within the ODW and compared them with a densified wood sample without oil impregnation. The densified wood without oil was fabricated using a similar delignification and densification procedure, except that the oil impregnation step was not conducted. The 1D SAXS analysis (fig. S3A) shows that ODW has a smaller intercellulose fibril distance of 2.8 nm than that of the densified wood without oil of 3.3 nm. This suggests that the oil may split the cellulose fibrils (fig. S3B), possibly due to the hydrophobic-hydrophobic interaction between the oil and the hydrophobic part of cellulose chains (*23*). Together with the SEM characterization of ODW, these results suggest that we successfully confined oil within the aligned 1D nanosized channels defined by the naturally anisotropic wood structure of ODW. Note that oil impregnation does not change the cellulose crystal structure in the OIP, ODW, and densified wood without oil, as shown by wide-angle x-ray scattering (WAXS) (fig. S4). Therefore, we do not need to consider the influence of the cellulose molecular arrangement on the dielectric, thermal, and mechanical properties of the materials.

### Evaluation and mechanism of the ODW’s electrical insulation

We next measured the dielectric properties of the ODW in comparison with the high-density (0.95 g cm^−3^) and low-density (0.71 g cm^−3^) OIP. In general, the relative permittivity of the insulation paper should be as low as possible so that more electrical stress can be distributed to the lignocellulosic insulation material rather than overstressing the insulating oil for power transformer design. Our measurements show that the 120-μm-thick ODW has a relative permittivity of 5.2 ± 0.078, slightly lower than the high-density OIP with a relative permittivity of 5.47 ± 0.31 (fig. S5). The slight difference may be due to the different raw materials used to prepare the ODW compared with those used for the high-density OIP. Meanwhile, the low-density OIP has a lower permittivity of 3.57 ± 0.25, mostly due to its higher oil fraction (fig. S5). This result is promising because the ODW displays suitable permittivity for transformer insulation applications.

We then evaluated the dielectric strength of the ODW under 60-Hz frequency voltage excitation following standard testing and analysis (see Materials and Methods for details). By sandwiching the sample between two electrodes and submerging in insulating oil, we can apply high voltage in an ac mode and determine when electrical breakdown occurs. After normalizing the breakdown voltages by the sample thickness, the dielectric strengths are obtained and statistically analyzed by fitting with the two-parameter Weibull distribution to determine a statistical dielectric strength following the International Electrotechnical Commission (IEC) 62539-2007 standard (*24*)

**Fig. 3.**
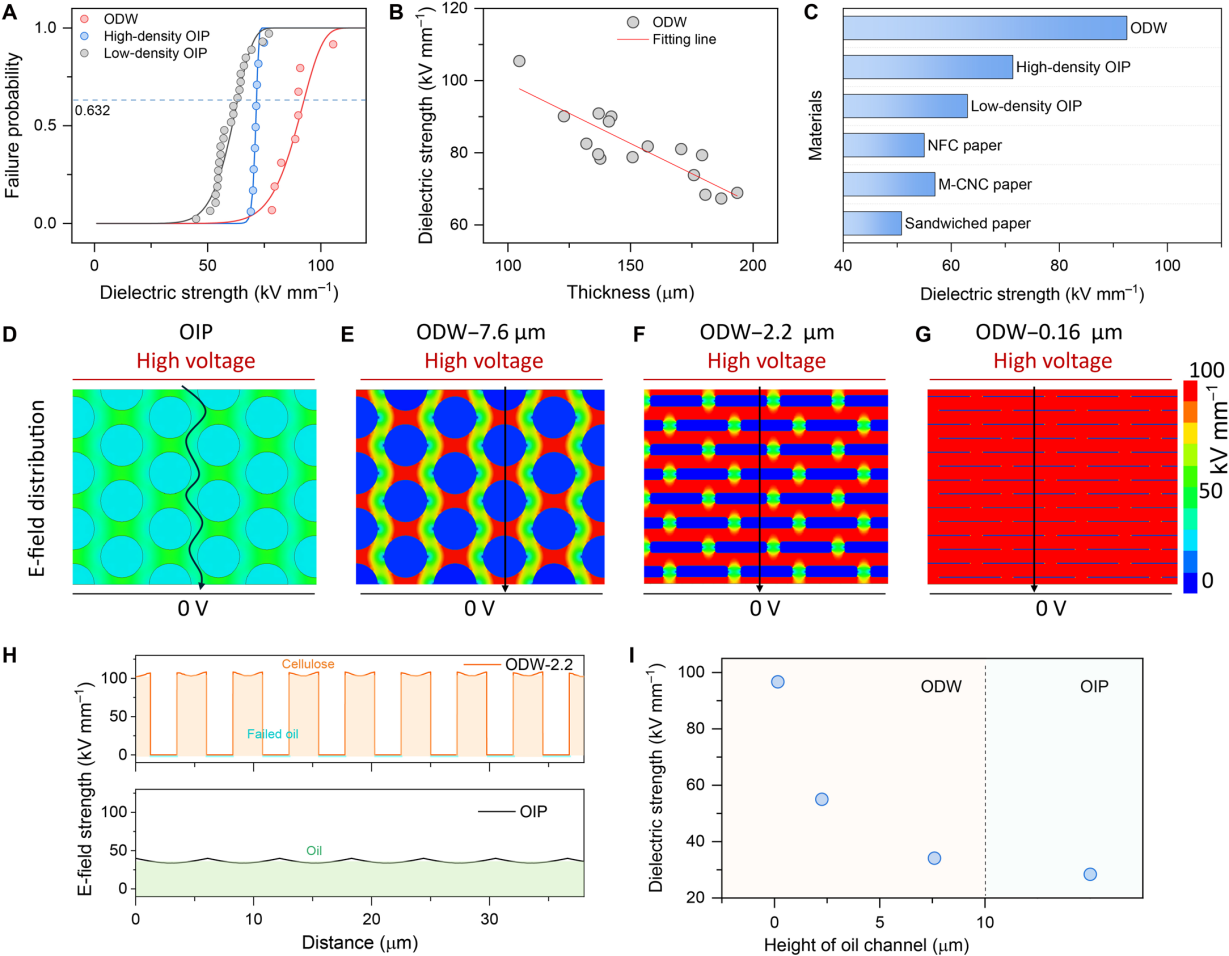
Measured and finite element simulation of the dielectric properties of the ODW. (**A**) ac breakdown strength comparison between ODW and low-density and high-density OIP (the estimated Weibull distribution plots are also provided in solid lines). The dielectric strength at the cumulative failure probability of 63.2% indicates the scale parameter of the materials. (**B**) Dielectric strength as a function of the ODW thickness. (**C**) Comparison of the dielectric strength of the ODW and other reported cellulosic insulation papers (*7*, *18*–*20*, *25*). NFC, nanofibrillated cellulose; M-CNC, modified cellulose nanocrystals. (**D** to **G**) The simulated E-field distribution of ODW and OIP, where voltages of 1 to 4 kV and 0 V are applied to the top and bottom surfaces of the insulation system, respectively. The black lines are typical breakdown pathways in each kind of structure. (**H**) E-field strength along the paths shown in the E-field color maps for OIP (D) and ODW–2.2 μm (F). The *x* axis describes the distance from the initial point of the dielectric breakdown path to its end, as shown in (D) and (F). (**I**) The simulated dielectric strength of the OIP and the ODW samples as a function of their oil channel height. Note that the OIP features a 3D interconnected oil structure without a well-defined size.

P(E)=1−e−(Eα)β (1)

where *E* is the measured dielectric strength; *P*(*E*) is the probability of failure at the breakdown strength less than or equal to *E*; α is the scale parameter, namely, the dielectric strength at the cumulative failure probability of 63.2%; and β is the shape parameter reflecting the data dispersion. From this fitting, we find that the ODW exhibits a record high dielectric strength of 105 kV mm^−1^ based on reported data of oil insulation papers (*7*, *18*–*20*, *25*) and a higher scale parameter of 92.6 kV mm^−1^ than both the low-density OIP (63.3 kV mm^−1^) and high-density OIP (71.6 kV mm^−1^), as shown in Fig. 3A. The dielectric strength of the ODW displays a negative correlation with the ODW paper thickness, corresponding to the oil channel height (Fig. 3B). For example, the dielectric strength of ODW with a thickness of 120 μm (i.e., with oil channel height of ~167 nm) is 92.6 kV mm^−1^ compared with 68 kV mm^−1^ for ODW with a thickness of 190 μm (i.e., with oil channel height of ~800 nm), demonstrating how the height of the oil channel notably affects the dielectric strength. Notably, the dielectric strength of the ODW is almost two times greater than the reported values for insulation papers (50 to 72 kV mm^−1^) (Fig. 3C) (*7*, *18*–*20*, *25*). Moreover, the greater dielectric strength of ODW than that of partially ODW sample (oil impregnation after densification) (fig. S6) verifies the effective oil impregnation of ODW achieved by our proposed process.

Furthermore, we also made ODW derived from balsa wood veneer (a hardwood with lower density; fig. S7) using the same procedure to demonstrate the versatility of this design approach in achieving nanosized 1D oil channels in highly aligned cellulose fiber structure. Using different wood species to produce the ODW could increase material selection flexibility for windings with small wire diameters to decrease the production cost. The ODW based on balsa wood has a dielectric strength of up to 90 kV mm^−1^, higher than both low- and high-density OIP, indicating the versatility of this material design method, which can be applied to various wood species. The balsa wood ODW also exhibits a negative correlation between the dielectric strength and the thickness of the densified material (fig. S7C), further suggesting the importance of the nanosized oil confined within the wood cell channels and the gaps between these channels, which are composed of highly aligned cellulose fibers, on the dielectric strength of the composite oil-cellulose material.

To examine the role of the size, ratio, and distribution of the insulating oil on the dielectric breakdown of these oil insulation paper composites, we used finite element simulations to analyze the electric field (E-field) distribution under breakdown conditions and compare the simulated dielectric strength among different scenarios. The simulation assumes no trapped air in the paper sample and thus omits air and its partial discharge. Only two constitutions, cellulose and oil, are considered. We investigated two kinds of oil structures: (i) micrometer-sized 3D interconnected insulating oil network with cellulose dispersed within the oil phase, mimicking the OIP case; and (ii) 1D insulating oil channels of varying size embedded within a continuous cellulose matrix, simulating the ODW (fig. S8, A to D). For the ODW, we modeled 1D oil channels with heights of 7.6 μm (ODW–7.6 μm), 2.2 μm (ODW–2.2 μm), and 0.16 μm (ODW–160 nm) to investigate the influence of oil channel size on the dielectric strength of the composite. By applying a voltage on these systems, the dielectric strength in the insulating oil and cellulose regions can be simulated on the basis of the dielectric constant of the two materials (3 and 6.1 for insulating oil and cellulose, respectively). For the OIP simulation, the insulating oil bears a high E-field strength and undergoes breakdown first, which enables a continuous breakdown path through the interconnected oil regions (Fig. 3, D and H), whereas the cellulose region remains intact as the E-field strength there is below the intrinsic dielectric strength of the cellulose (100 kV mm^−1^). As a result, the equivalent dielectric strength of the entire OIP system is only about 28.4 kV mm^−1^ (Fig. 2I).

Meanwhile, given the isolated oil structure of the ODW, our modeling of the electrical breakdown of the ODW followed a two-step procedure: (i) Breakdown occurs first in the isolated 1D oil under a relatively low excitation voltage (fig. S8, E to G); and (ii) the lignocellulose fibers subsequently undergo electrical breakdown due to the E-field redistribution after the oil failure, which leads to the complete breakdown of the ODW composite, with a continuous breakdown pathway through the cellulose region and initially failed oil region, as shown in Fig. 3H. Thus, under this simulation methodology, ODW–7.6 μm shows a dielectric strength of 34.1 kV mm^−1^ (Fig. 3, E and I). By adjusting the shape of the 1D oil channel and reducing its height from 7.6 to 0.16 μm, the simulated dielectric strength increases from 34.1 kV mm^−1^ (ODW–7.6 μm) to 55 kV mm^−1^ (ODW–2.2 μm) and then to 96.7 kV mm^−1^ (ODW–160 nm) (Fig. 3, E to G and I), or the two reduced oil channels enhanced 1.6 and 2.8 times of the value for ODW–7.6 μm, respectively. In addition, the dielectric strengths of the three ODWs with different channel sizes are all greater than that of OIP, which is only about 28.4 kV mm^−1^. Note that the smaller simulated dielectric strength of OIP compared with the measured one is ascribed to the large fraction of connected oil in the OIP structure model. In contrast, the 1D isolated oil channel morphology of the ODW can effectively improve the dielectric strength of the composite. Furthermore, decreasing the channel height of the 1D oil to nanometer scale can substantially increase the overall dielectric strength of the insulation system, which is close to 100 kV mm^−1^, the limit of cellulose.

### Mechanical and thermal properties of ODW

**Fig. 4.**
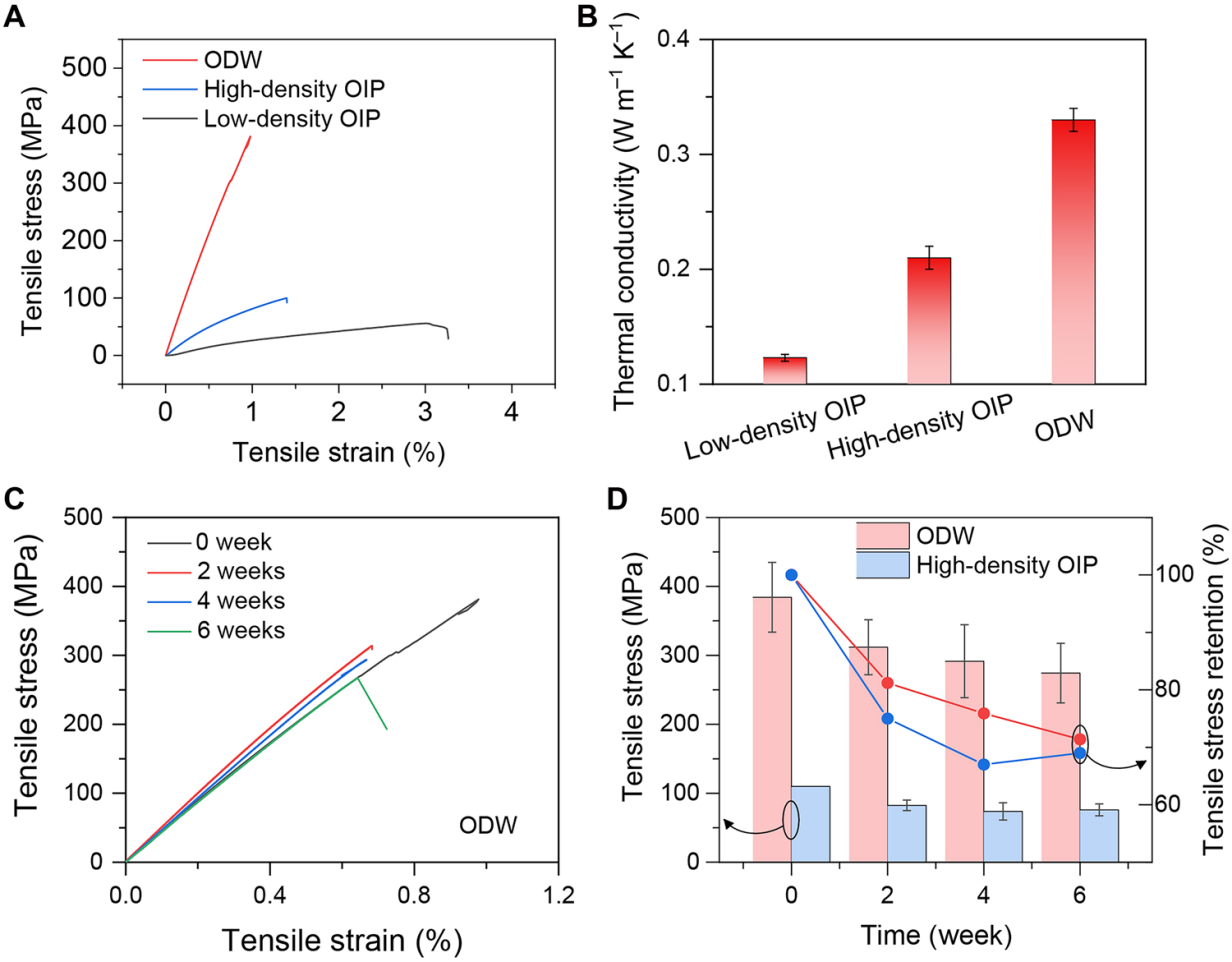
Mechanical, thermal conductivity, and thermal aging properties of the ODW. (**A**) Tensile stress-strain curves of the ODW and low and high-density OIP. (**B**) Through-plane thermal conductivities of the ODW and low- and high-density OIP. (**C**) Tensile stress-strain curves of the ODW aged at 150°C for various durations of time, ranging between 0 and 6 weeks. (**D**) The corresponding tensile stress change of the ODW and high-density OIP after various thermal aging times.

In addition to the dielectric strength, we also measured the mechanical strength and thermal conductivity of the ODW–160 nm to determine its durability for practical applications. Mechanical testing revealed the ODW has a tensile strength of 384 MPa, which is much higher than that of conventional low-density OIP (56 MPa) and high-density OIP (100 MPa) (Fig. 4A). We attribute this improvement to the highly aligned cellulose fiber chains in the ODW with the retaining of integrity wood physical structure, as well as the substantial increase in hydrogen binding due to substantial densification after partial lignin removal. Thermal conductivity is another essential parameter for the insulation paper as it lowers the material’s operation temperature and extends its life. The through-plane thermal conductivity of the ODW was 0.33 W m^−1^ K^−1^, higher than the high-density and low-density OIP with values of 0.21 and 0.12 W m^−1^ K^−1^, respectively (Fig. 4B). The enhanced thermal conductivity of ODW over OIP may result from the larger cellulose content, the formation of a continuous solid-phase heat transport network via direct cellulose-cellulose contact arising from the densification process, and the highly aligned fiber structure that may help reduce interfacial phonon scattering (*26*–*28*).

Slower thermal degradation is also important to consider since the thermal aging of insulation paper shortens the fiber chains and weakens the interaction forces between them, thereby reducing the mechanical and dielectric performance of the material, which eventually damages power transformers by thermomechanical or dielectric breakdown (*16*, *29*). To mimic the operation condition of insulation paper inside the power transformers, we performed an accelerated thermal aging process under inert atmosphere at 150°C based on IEEE C57.100-2011 testing standard (*30*). The tensile strength was then monitored over 6 weeks to evaluate the degradation of the insulation paper (fig. S9). As the aging time increases, the tensile strength of the ODW gradually reduces from 384 to 274 MPa at 6 weeks (28.6% reduction; Fig. 4C), likely due to the hydrolytic breakdown and thermal scission of the β-1,4 glycosidic bond in cellulose chains under thermal treatment (*15*). The high-density OIP displays a similar decreasing trend, from 110 to 76 MPa at 6 weeks (31% reduction) (Fig. 4D). The slightly higher retention of tensile strength suggests that the ODW is slightly more durable than conventional OIP. However, the notably greater mechanical strength of the ODW compared to the commercial OIP after aging for 6 weeks (i.e., 274 MPa versus 76 MPa) demonstrates ODW retains superior mechanical strength after thermal degradation test. These results demonstrate the promising properties and stability of the ODW, including high mechanical strength, excellent thermal stability, high through-plane thermal conductivity, and high dielectric strength with acceptable permittivity, suggesting it as a promising alternative for application in power transformers (table S1).

### Large-scale ODW demonstration

**Fig. 5.**
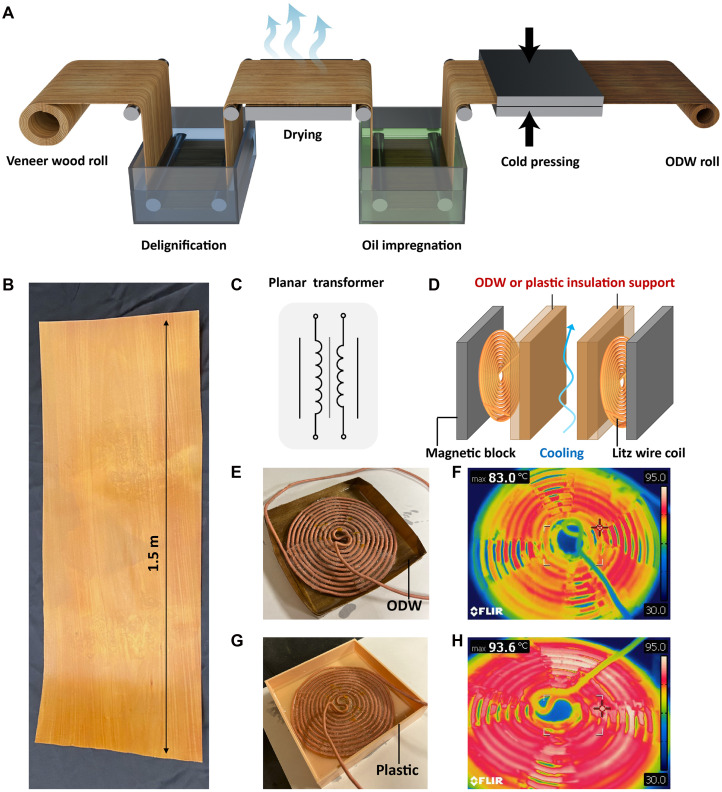
Demonstration of large-scale ODW. (**A**) Schematic diagram of the roll-to-roll procedure to make ODW by continuous delignification, drying, oil impregnation, and densification process. (**B**) A large-size sheet of ODW with dimensions of 1.5 m in length and 0.4 m in width. (**C**) Circuit symbol diagram of a planar transformer. (**D**) Schematic of the planar transformer. The planar transformer is composed of two insulation boxes as the sending and receiving sides, where the space between the boxes is applied as the cooling and insulation channels. (**E**) Photo of the half planar transformer and (**F**) the measured temperature contour, where the insulation box is made with ODW. (**G**) Photo of the half planar transformer and (**H**) the measured temperature contour, where the insulation box is made with plastic. The planar transformers have 35-W winding loss.

Fabricating the ODW from natural wood veneer can be readily scaled as the synthesis is compatible with a roll-to-roll continuous process (Fig. 5A) and could be adapted to fit in current insulation paper production lines with some modifications. To demonstrate the scalability of our proposed approach, we successfully made large-size ODW samples with lengths of over 1.5 m (Fig. 5B). The proposed ODW is also flexible, meeting the flexibility requirements to wrap over the copper bars (windings) in power transformers, as demonstrated using the continuous and seamless winding of ODW on an aluminum rod (fig. S10).

To further illustrate the application capability of ODW, we fabricated a planar transformer (Fig. 5C), which can be applied in a modular multilevel converter, a key component in power transmission, energy storage, and industrial motor drives (*31*, *32*). For a planar transformer design (Fig. 5D), the ideal material for the insulation support should have high through-plane thermal conductivity, dielectric strength, and sufficient mechanical strength. As a proof of concept, we fabricated square insulation boxes composed of ODW, which were mechanically strong enough to hold the ferrite magnetic blocks and coils of the planar transformer, with a total weight of ~5 kg (Fig. 5E). We also made boxes from conventional plastic composed of acrylonitrile butadiene styrene polymer to evaluate as a control sample (Fig. 5G). By conducting a current of 37 A in the transformer’s Litz wire coil to mimic the operation condition (heat generation) and applying forced air cooling underneath the support interface (heat dissipation) (Fig. 5D), the temperature distribution of the Litz wire coil was monitored by an infrared camera to evaluate the heat dissipation capability of the insulation support. After operation for ~10 min, the maximum temperature of the setup using the ODW insulation box stabilized at 83.0°C, which is lower than that of the plastic insulation setup (93.6°C) under the same test conditions (Fig. 5, F to H, and fig. S11). We primarily attribute the ~10°C hotspot temperature reduction of the ODW due to its higher thermal conductivity (0.33 W m^−1^ K^−1^) compared to the plastic (0.15 W m^−1^ K^−1^), which we further verified by the finite element analysis (fig. S12), where we found a temperature reduction of ~15°C for the setup using ODW compared to the plastic one. These results demonstrate the feasibility of applying the proposed ODW in a real transformer.

## DISCUSSION

In this work, we have developed and demonstrated densified oil-impregnated wood veneers as a promising insulation paper for power transformer applications. The naturally anisotropic structure of the wood promotes the formation of nanosized oil channels isolated by the higher dielectric strength cellulose—a structure that increases the composite’s overall dielectric strength by disrupting the electrical breakdown pathways as suggested by finite element simulation. The ODW can be fabricated by a continuous process, which has the potential for large-scale fabrication by a roll-to-roll setup. The process converts the porous wood into densified wood veneer, in which the shrunken lumina (166 ± 87 nm) and gaps between neighboring cells are filled with insulating oil. Because of the densified and aligned lignocellulose matrix and isolated nanosized oil channels, the ODW achieves a high dielectric strength of up to 105 kV mm^−1^, a record among lignocellulose-based oil-impregnated papers. In addition, the ODW displays a high mechanical strength of up to 384 MPa and through-plane thermal conductivity of 0.33 W m^−1^ K^−1^. The ODW also exhibits enhanced thermal stability based on thermal aging tests, with a mechanical strength of 274 MPa after 6 weeks of thermal degradation at 150°C. The excellent comprehensive performance of the ODW demonstrates itself as an alternative insulation material for power transformers. In addition, this ODW veneer-based structure suggests a general strategy for enhancing dielectric performance, in which 1D nanoscale channels of air-excluding medium could be impregnated with epoxy to form a solid-state hybrid insulation system for application in dry-type transformers, motors, printed circuit boards, etc.

## MATERIALS AND METHODS

### Materials and chemicals

Basswood (500 μm in thickness) and balsawood veneer (500 μm in thickness) were purchased from Specialized Balsa Wood LLC. Sodium hydroxide (reagent grade, Sigma-Aldrich) was used for the partial removal of lignin from wood. FR3 fluid (the natural ester insulating oil) was purchased from local distributors of Cargill Corp. Low-density (0.71 g cm^−3^) and high-density (1 g cm^−3^) commercial insulation paper was donated by Weidmann Electrical Technology AG.

### Fabrication of the ODWs and densified wood without oil impregnation

First, the wood veneers were immersed and boiled in 5 wt % NaOH solution for 2 hours to partially remove lignin and hemicelluloses, followed by washing with excess deionized water to thoroughly remove the residual chemicals. Next, the partially delignified wood veneers were dried by a freeze-dryer overnight and then placed under vacuum at 105°C for 3 to 4 hours, followed by impregnation with insulating oil (moisture-free and gas-free) under vacuum at room temperature for 12 to 24 hours. The insulating oil was previously dried at 115°C under vacuum for 18 hours to thoroughly remove residual moisture and gas. Last, the oil-impregnated wood veneers were compressed using a press (YLJ-HP88V-250, MTI Corporation) with a controlled pressure (1 to 7 MPa) overnight to obtain the ODW samples. Note that the selection of the ester fluid was based on the industry practice of phasing out mineral oil in transformers in favor of ester-based liquids to reduce flammability hazards.

For the densified wood without oil impregnation, the same delignification process was applied, namely, boiling the wood veneer in the 5% NaOH for 2 hours and washing with water. The resulting partially delignified wood veneer was then freeze-dried overnight and dried under vacuum (same as the ODW drying procedure). Next, the material was compressed at 7 MPa at room temperature, resulting in the densified wood without oil impregnation.

### Characterizations

SEM was conducted at 5 kV on a Hitachi SU-70 microscope. Before SEM imaging, oil was extracted from the ODW and OIP samples with hexane to reveal the underlying structure. The native cellulose architecture remains intact throughout this process due to its insolubility in hexane. The tensile strength tests were conducted according to the ASTM D3379-75 standard (*33*) using an Instron 3365 universal testing system with a load capacity of 30 kN at a crosshead speed of 5 mm min^−1^. At least five replicate samples were measured for ODW and OIP.

### SAXS and WAXS measurements

SAXS and WAXS measurements were conducted at the Soft Matter Interfaces (SMI) beamline (12-ID) of the National Synchrotron Light Source II, Brookhaven National Laboratory. The experiments used a monochromatic x-ray beam with an energy of 16.1 keV (corresponding wavelength λ ≈ 0.77 Å) and an energy resolution of Δ*E*/*E* = 0.01%. The beam was focused to dimensions of 200 μm (horizontal) by 30 μm (vertical) at the sample position. Scattered x-rays were detected using a PILATUS3 900 kW detector (Dectris, Switzerland), featuring a 1475 by 619 array of 0.172 mm–by–0.172 mm pixels, positioned at a sample-to-detector distance of 275 mm.

Thin-film samples (~1 cm by 0.5 cm) were sandwiched between two aluminum bars and mounted in a custom-built humidity-controlled chamber attached to a hexapod stage. Transmission WAXS patterns were collected using the PILATUS3 900 kW detector. To ensure statistical reliability, measurements were obtained at three distinct positions on each sample, with an exposure time of 1 s per position. The detector was rotated along an arc to access a wide range of scattering vectors (*q*). The resulting series of 2D diffraction patterns were subsequently stitched together and reduced to 1D *q*-*I*(*q*) profiles using an in-house developed Python script. Calibration of the scattering angle (2θ) and *q*-space conversion were performed using a silver behenate powder standard [*d*(001) = 58.38 Å].

### Thermal conductivity test

Through-plane thermal conductivity was measured using a Hot Disk TPS 2200 S instrument based on the transient plane source method. The Isotropic Module and the Thin Film Module with Pyrex glass background were used for the measurement of ODW with a thickness of 120 μm and commercial OIP, respectively. For all measurements, a Kapton sensor was sandwiched between two identical samples, serving as both a heat source and a temperature detector. A 9-lb (4.08-kg) weight was placed on the top of the samples, which was used to ensure satisfactory contact between the sensor and samples. During measurement, an electrical current heated the sensor, and the resulting temperature rise was recorded as heat diffuses into the specimens. The thermal conductivity was then calculated by the system software from the recorded temperature response. The reported thermal conductivity was the average of at least three measurements.

### Thermal aging test

To mimic the environment inside the power transformers, the thermal aging test was performed in a self-designed test vessel made of stainless steel. Insulation paper, oil, and copper were loaded into the vessel with a fixed oil to paper and oil to copper ratio, which were all defined in the ASTM D2413-16 standard (*34*). Vessels were properly cleaned and sealed, while the insulating oil was dried and degassed before use. Pure nitrogen was introduced to replace the air in the headspace of the vessel, which matched the internal conditions of some real transformers and facilitated producing consistent aging data. The insulation paper samples were heated at 150°C with controlled durations within the vessel to simulate thermal aging. At least 10 samples with the same treatment were tested to obtain an average value.

### Dielectric breakdown test and its statistical analysis

The breakdown test setup and procedure follow the IEC 60243-1 standard (*35*). A commercial 60-Hz high-voltage power supply with a constant voltage ramping rate of 750 V s^−1^ was applied in the experiments. A pair of parallel plate electrodes in round shape were fabricated with a diameter of 25 and 75 mm for the high voltage (HV) electrode and ground electrode, respectively, with a corner rounding radius of 3 mm. An epoxy encapsulant was carefully applied along the rounded edges of the HV electrode to avoid severe arcing in the oil gap between the rounded corner of the HV electrode and the tested sample. The sample was sandwiched between the parallel electrodes, and a Fowler Quadra test indicator was used for thickness measurement. The reported average thickness was determined from at least five replicate measurements.

On the basis of the recorded breakdown voltage and average thickness of each sample, the breakdown strength of every test sample can be estimated based on Equation 1.

To estimate the two parameters of the Weibull distribution, we place the breakdown samples in order from smallest to largest and assign them a rank from *i* = 1 to *i* = *k*. Then, a simple approximation for the probability of failure is listed as follows

$$F\left(i,k\right)\approx \frac{i-0.44}{k+0.25}\times 100\%$$ (2)

### Relative permittivity test

Relative permittivity was determined by measuring the capacitance introduced by the oil-impregnated insulation coupon between two parallel plate electrodes. The electrode system was the same as the one used in the dielectric strength test. The applied insulating oil has a relative permittivity of about 3 to 3.2 (*10*). We used an Instek LCR meter with 0.1-pF resolution and 0.05% accuracy to measure the capacitance. The test frequency ranged from 10 to 10,000 Hz. At least 10 samples were tested to get an average value.

The relationship between the capacitance *C* and the relative permittivity ε_r_ of the sample under testing is described by

$$C=\varepsilon_{0}\varepsilon_{r}\pi r^{2}/d$$ (3)

where C is the measured capacitance; *r* is the radius of the top electrode; *d* is the interelectrode spacing after the test coupon is inserted; $\varepsilon_{r}$ is the relative permittivity of the test coupon; and $\varepsilon_{0}$ is the permittivity of free space, which is 8.8542 F m^−1^.

### Demonstration of a planar transformer

The planar transformer consisted of two identical boxes as the sending and receiving sides of the wireless power transfer system. The ODW insulation box (11 cm by 11 cm in length and width and 1.2 cm in height) was made by hand-bending the ODW, while the plastic box was made by 3D printing acrylonitrile butadiene styrene. To mimic the setup of a typical planar transformer, inside each insulation box, we added a coil made of Litz wire with a ferrite magnetic block over the coil, which was then sealed with silicone gel to the box. By conducting a current of 37 A in the Litz wire coil to mimic the operation condition (heat generation) and applying forced air cooling underneath the support interface (heat dissipation), the heat dissipation capability of the insulation support system was evaluated by monitoring the temperature distribution of the Litz wire coil.

### Thermal profile simulation in the planar transformer

The finite element analysis method was applied for this simulation using the commercial software Ansys. The model of planar transformer includes an insulation box, which contains the coil and silicone gel with thermal conductivity of 385 and 0.2 W m^−1^ K^−1^, respectively. The coil was simplified using a copper disk structure in simulation with a similar area and thickness as the Litz wire coil. The parameters for simulation were as follows: an ambient temperature of 25°C; a convective coefficient of 150 W (m^2^ °C)^−1^ was applied to the bottom interface of the box; and the winding loss was 35 W, evenly distributed in the coil. An automatic adaptive mesh method was applied for the mesh setting. The four walls of the box (except the top and bottom walls) were considered thermally insulated.

### Finite element simulation for breakdown strength estimation

The finite element method was applied to predict the E-field distribution within the cellulose-oil composite material by solving Eq. 4 (*36*). A two-step procedure was used to identify the breakdown path and estimate the breakdown strength of the material. Specifically, in the first step, the entire mixture of cellulose and oil was analyzed with an increase in the applied voltage. Once the E-field strength exceeded the breakdown strength of the insulating oil regions, the oil regions were treated as breakdown and could no longer hold the electrical stress. In the second step, the E-field redistribution analysis was applied after the oil failure. The second step analysis may iterate with an increase in voltage if the E-field strength after the redistribution analysis does not exceed the cellulose dielectric strength. Thus, the breakdown strength of the material can be obtained using Eq. 5

$$\nabla \cdot \varepsilon \vec{E}=\rho_{f}$$ (4)

∣E→breakdown∣=Vbreakdownτ (5)

where $\;\vec{E}$ is the E-field vector, ρ_f_ is the free volume charge, and ε is the permittivity of the material. $|{\vec{E}}_{\text{breakdown}}|$ is the estimated breakdown strength, $V_{\text{breakdown}}$ is the applied voltage when the breakdown of cellulose can be identified, and τ is the thickness of the material. No free volume charge is assumed in the simulation. The dielectric constants for cellulose and oil are considered as constant value of 6.1 and 3, respectively, and their estimated dielectric strengths applied in this simulation are 100 and 32.5 kV mm^−1^, accordingly (*10*, *37*).

Simplified geometric models of the cellulose-oil system were established on the basis of the measured characteristic oil height of ODW and the fiber diameter in the OIP, where the oil channel and fiber were arranged in a dense packing. For all simulations, high voltage and ground conditions were applied to the top and bottom geometric boundaries, respectively, while the symmetric boundary conditions were assigned for both the left and right geometric boundaries. An adaptive mesh method with fine quality was selected to obtain stable and accurate results. For convergence, at least one converged iteration was required with a relative error of <1% compared to the previous iteration.
