# Supplementary material for: Oil-impregnated densified wood veneer with high electrical insulation enabled by nanosized oil channels
Source: Sci Adv. 2026 Jun 10;12(24):eaed5744. doi: 10.1126/sciadv.aed5744 (PMC13251868; doi:10.1126/sciadv.aed5744)
Supplement: Supplementary file 1 — Figs. S1 to S12 Table S1 [file sciadv.aed5744_sm.pdf]

Science Advances
AAAS

Supplementary Materials for

Oil-impregnated densified wood veneer with high electrical insulation enabled by nanosized oil channels

Meiling Wu *et al.*

Corresponding author: Liangbing Hu, liangbing.hu@yale.edu

Sci. Adv. **12**, eaed5744 (2026)
DOI: 10.1126/sciadv.aed5744

This PDF file includes:

Figs. S1 to S12
Table S1

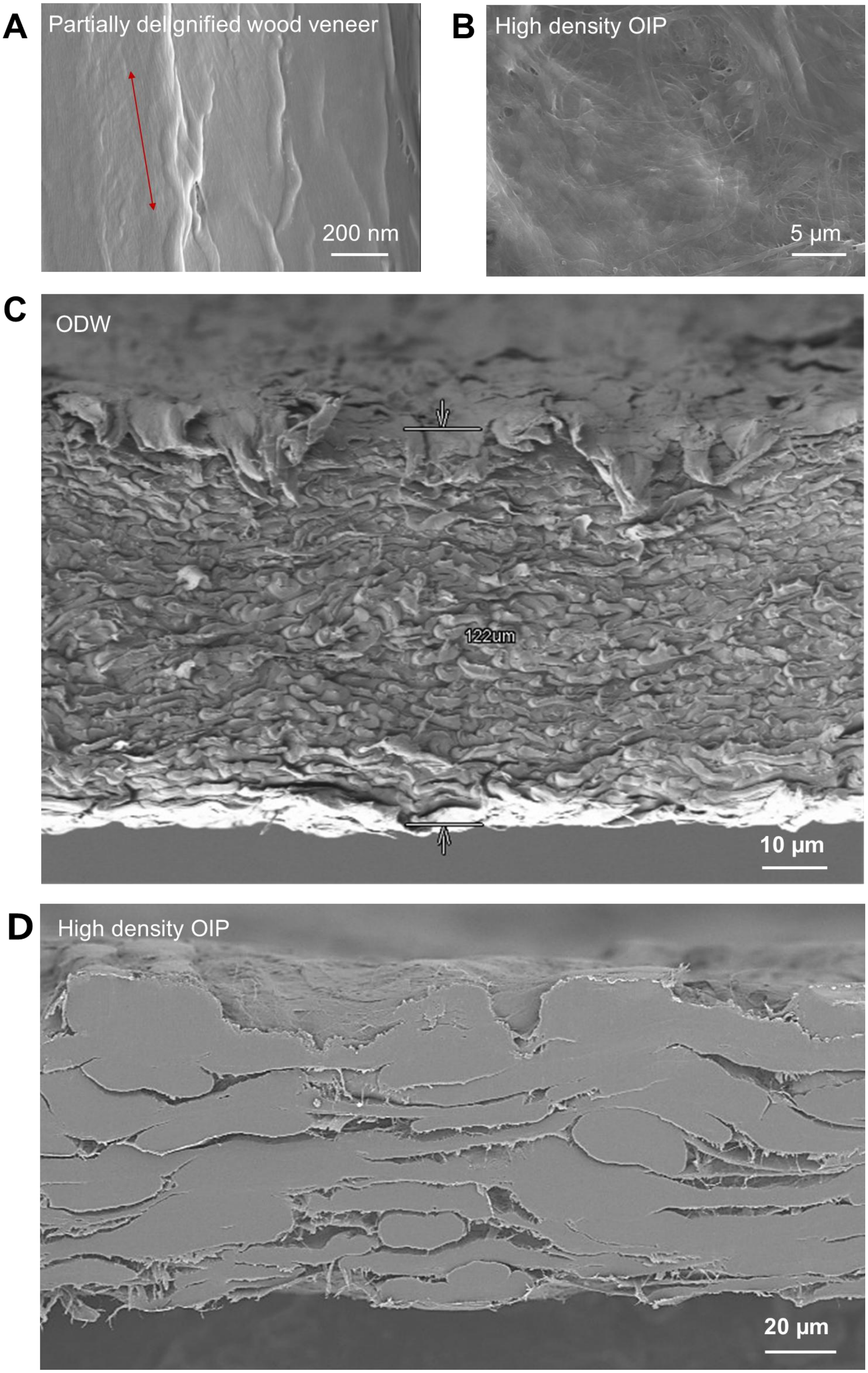

Fig. S1. Morphology of wood-based samples and OIP. SEM images of (**A**) the partially delignified wood veneer, with the growth direction indicated by the red arrows. The nanofibers shown in (**A**) are highly aligned along the

growth direction. (**B**) Surface morphology of the high density OIP, where cellulose fibers are distributed randomly and with significantly less alignment compared to the wood veneer. (**C**) Cross-sectional morphology of the ODW with thickness of ~120 μm. (**D**) Cross-sectional morphology of high density OIP. Part of fig. S1c and S1d are also shown in Fig. 2F and Fig. 2I of the main text, respectively. Larger views are presented herein to clearly illustrate the cross-sectional morphologies of the ODW and OIP samples.

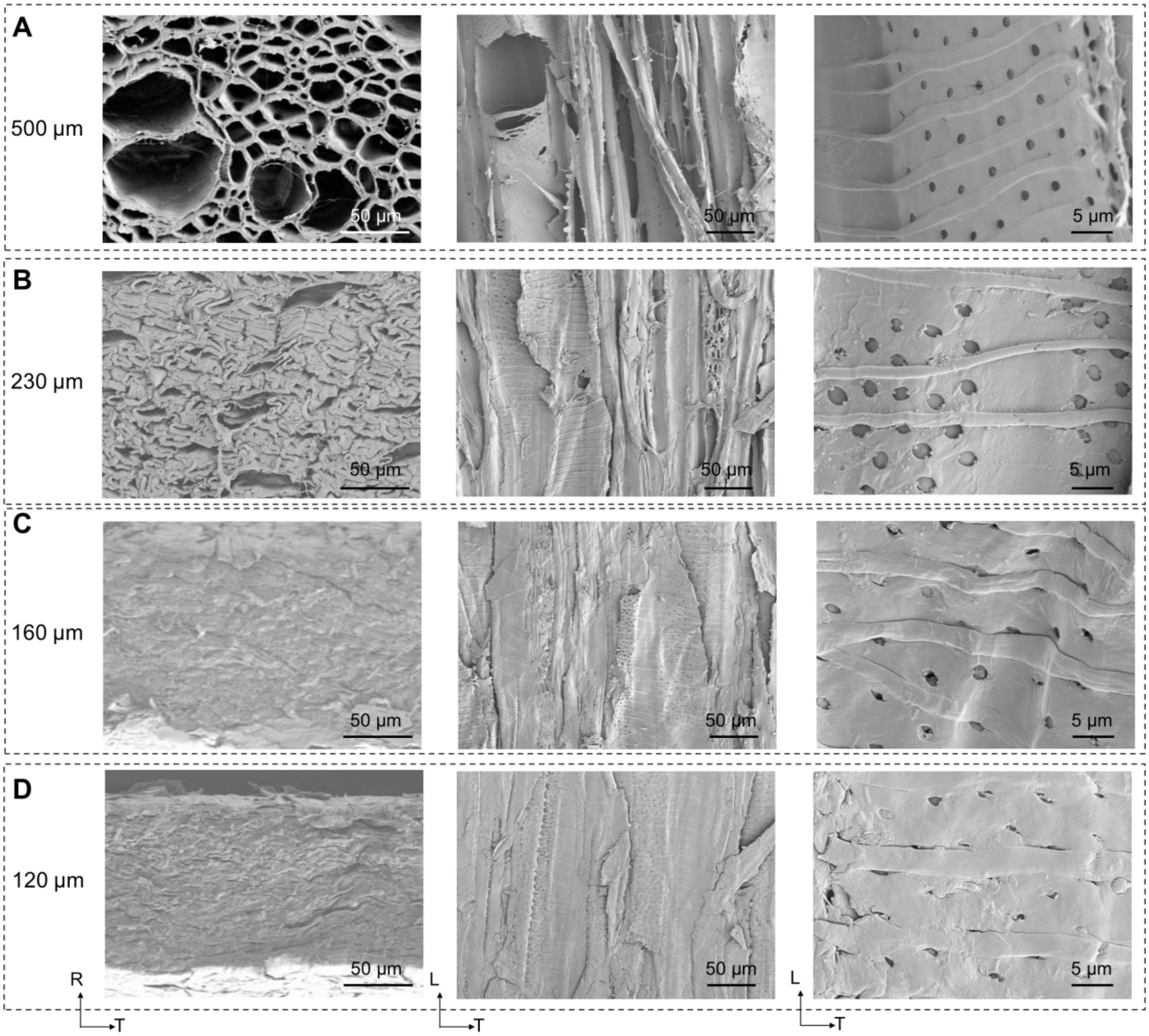

Fig. S2. ODW morphology with different densified thicknesses. Cross-sectional SEM images (perpendicular to the tree growth direction) and SEM images (along the tree growth direction) of ODW fabricated by controlling the densification pressure from 1 MPa to 7 MPa to achieve different veneer thicknesses of (**A**) 500 µm; (**B**) 230 µm; (**C**) 160 µm, and (**D**) 120 µm. With the increasing degrees of densification, the wood channels gradually collapse to a dense laminated structure. Round pits exist on the cell walls and connect to nearby cell walls. With the increasing degrees of densification, the cell walls collapse, and the pits are gradually densified with decreasing diameter. The middle images of fig. S2a and S2d are also shown in Fig. 2D and 2G of the main text, respectively. They are reused herein to facilitate a clear comparison of the morphology of samples with different degrees of densification. With the increasing degrees of densification, the wood channels gradually collapse to a dense laminated structure. Round pits exist on the cell walls and connect to nearby cell walls.

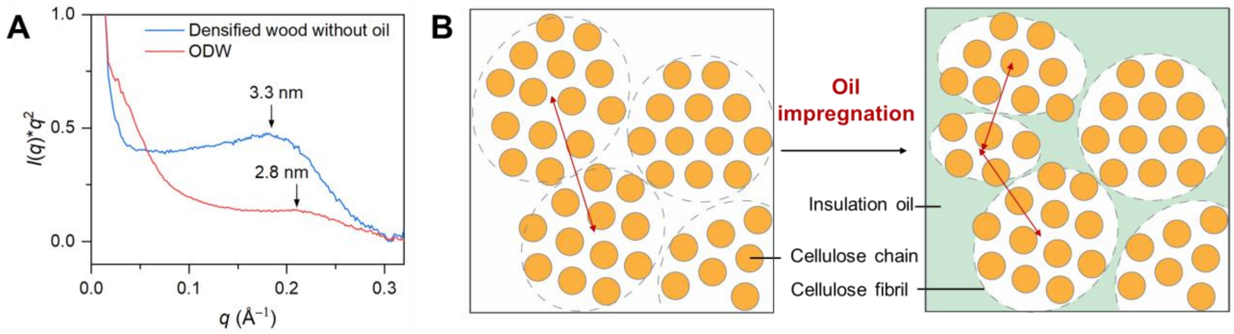

Fig. S3. SAXS profile and the predicted oil distribution in ODW. (**A**) 1D-SAXS profiles of the ODW and densified wood without oil. (**B**) Schematic structure of the ODW with oil impregnation. Cellulose fibrils (indicated by the dashed circles) are made by multiple cellulose chains (the orange circles). The distances between two cellulose fibrils are indicated by the double arrows.

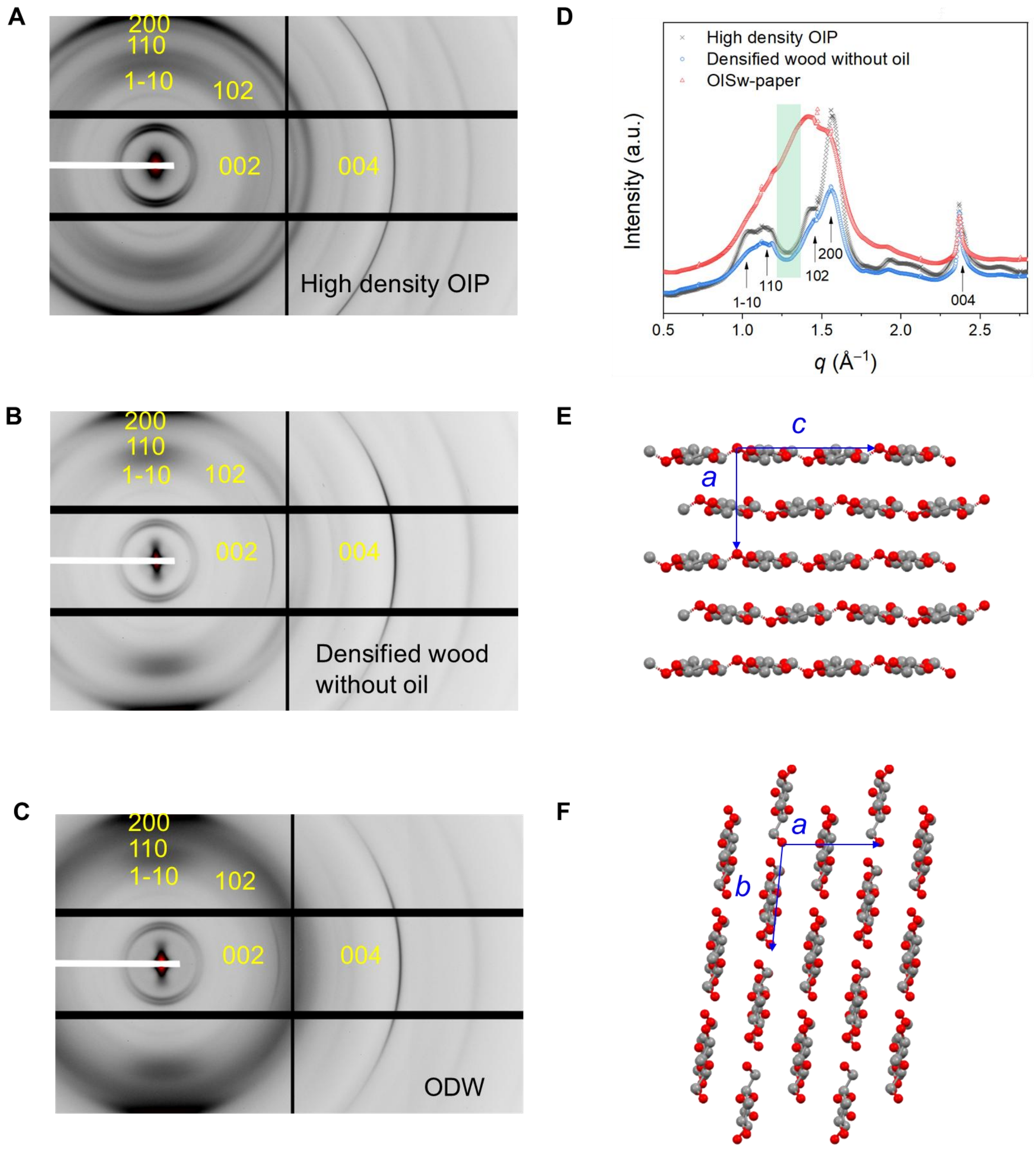

Fig. S4. WAXS measurements. WAXS pattern of (**A**) high density OIP, (**B**) densified wood without oil and (**C**) ODW. Major diffraction patterns or peaks are indexed in Fig. S4a-c, including (1–10), (110), (102), (200), (002), and (004). These peaks are consistent with the characteristic peak of cellulose I_{β}, which has a monoclinic unit cell with a space group of $P112_1$. The unit cell is a = 7.784 Å, b = 8.201 Å, c =10.380 Å and α = 90°, β = 90°, γ = 96.55°. (**D**) The corresponding 1D profiles of the change of the Lorentzian-corrected intensity against the scattering vector q (q= $(4\pi/\lambda)\sin\theta$, with λ and θ being the X-ray wavelength (1.542 Å) and half of the scattering angle, respectively). The scattering intensities are deduced from the equator direction of the 2D WAXS patterns. (**E-F**) Molecular structure of the cellulose crystal in the (ac) and (ab) planes.

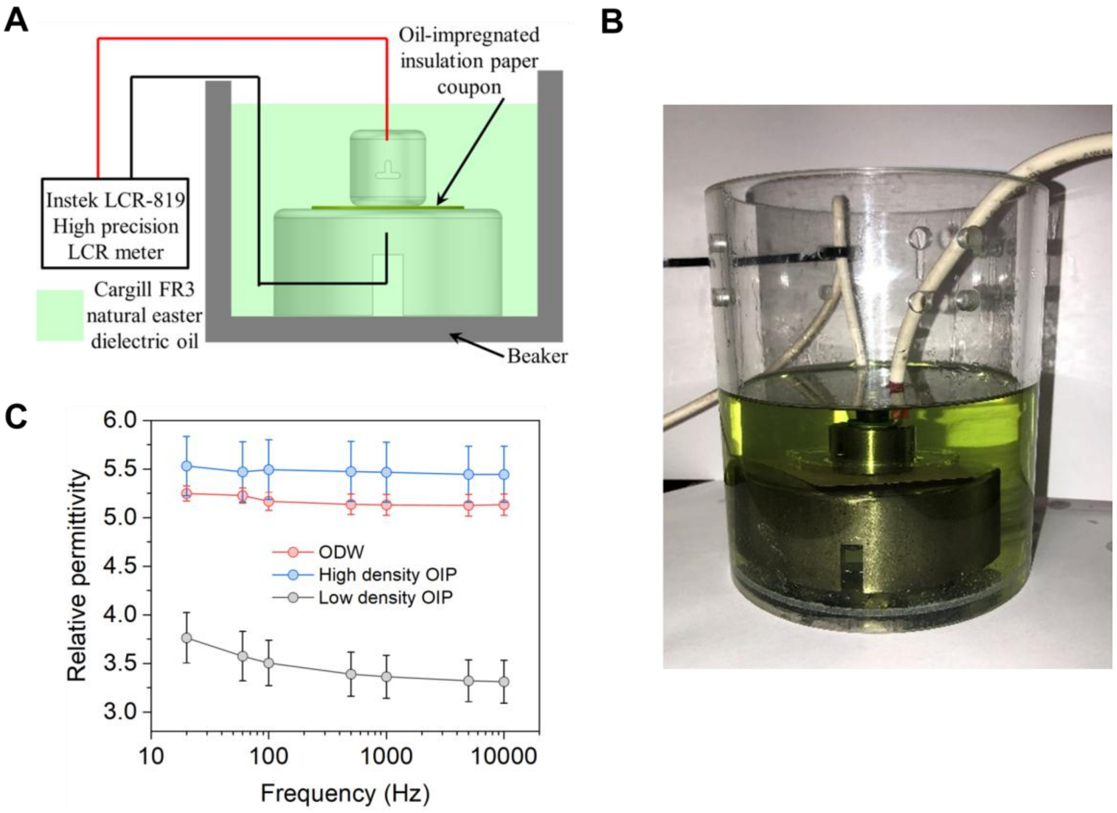

Fig. S5 Relative permittivity measurements. (**A**) Diagram of the setup for the relative permittivity testing of the ODW and high and low density OIP samples, which were measured between two parallel plate electrodes in round shape with diameter of 25 mm and 75 mm for the HV electrode and ground electrode, respectively. (**B**) Photo of the electrodes with the test sample submerged in transformer oil. (**C**) The relative permittivity of the ODW and high and low density OIP. Error: Standard Deviation.

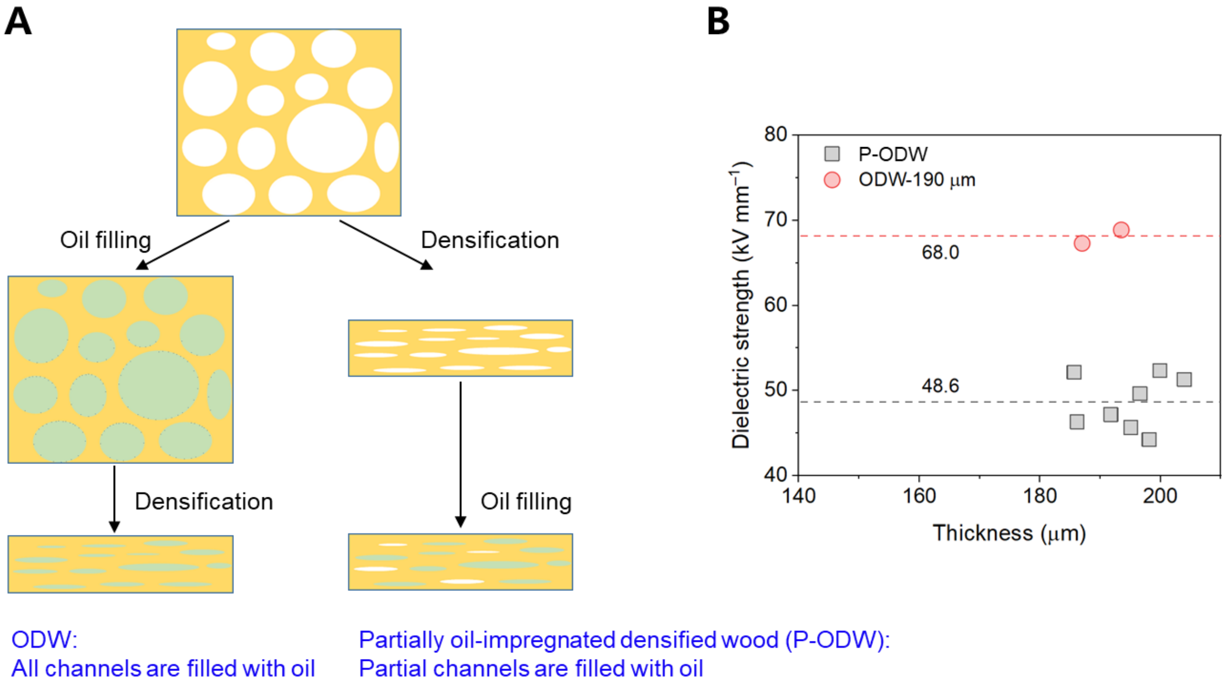

Fig. S6 Dielectric strength of ODW and P-ODW. (**A**) Schematic procedure to make ODW and P-ODW and (**B**) their dielectric strength at similar thickness. P-ODW is made by oil absorption after densification, which is likely to leave some channels incompletely filled due to trapped air in nanosized pocket. Thus, the P-ODW shows lower dielectric strength (48.6 kV mm^{-1}) than ODW (68.0 kV mm^{-1}) at similar thickness of 190 μm.

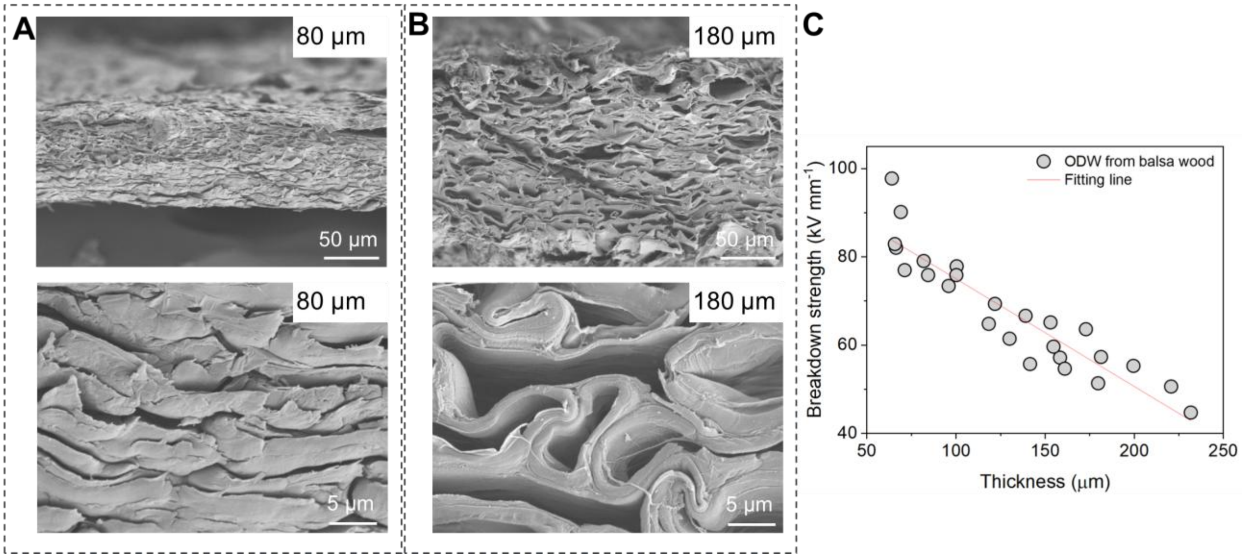

Fig. S7. Morphology and dielectric breakdown strength of ODW derived from balsa wood. Cross-sectional SEM images of balsa wood ODW with thicknesses of (**A**) 80 µm and (**B**) 180 µm. (**C**) Dielectric strength as a function of the thickness of the balsa wood ODW.

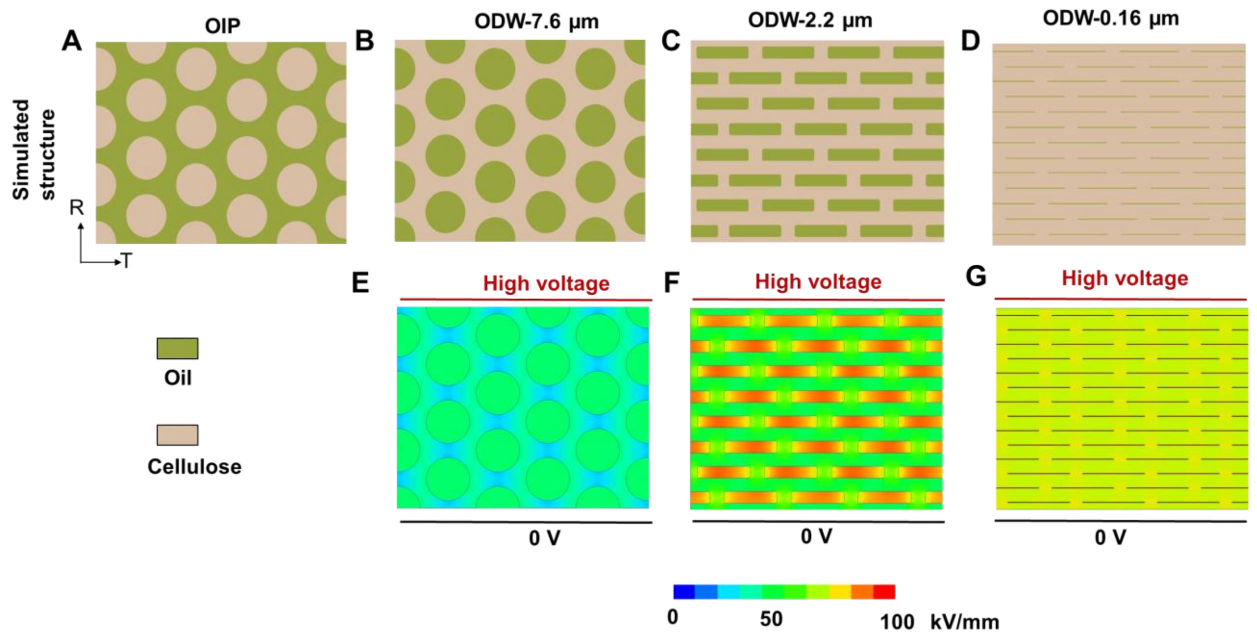

Fig. S8. Simulation models and the simulated electric field distribution. (**A-D**) Structures for the simulation of ODW and OIP. (**E-G**) The simulated electric field distribution in the first step of electric field distribution analysis of the ODW, where a voltage of 1–4 kV and 0 V are applied to the top and bottom surface of the composite paper, respectively. For the ODW samples, in the first step of the electric field distribution simulation, we can observe the electric field in the oil region is higher than the cellulose region, thus partial breakdown will occur in the oil regions and therefore the oil regions can be treated as failure and no longer hold the voltage stress after the E-field strength in the oil is over its intrinsic dielectric strength of 32.5 kV mm^{-1}.

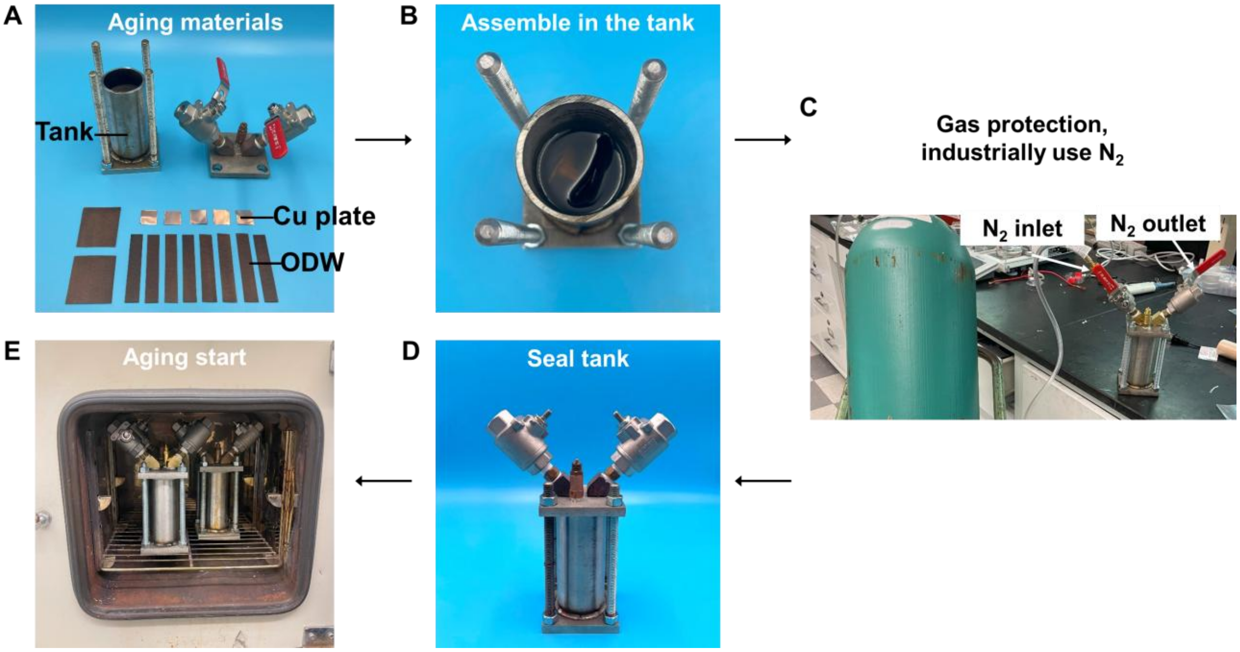

Fig. S9. **Procedure and setup for the thermal aging test.** (**A**) Components of the setup for the thermal aging test, which consisted of a tank, tank lid, copper conductor, and ODW (166 nm oil channel height, Length: 80 mm, Width: 15 mm). (**B**) All aging materials including the copper conductor and ODW were immersed in transformer oil. The volumetric ratio of oil to insulation paper or ODW was 17.6. (**C**) Industrially used N_2 is applied as a protective gas to eliminate oxygen-induced oxidization of the insulation paper. (**D-E**) Sealed tanks are placed in the oven and aged with user-defined temperature and duration.

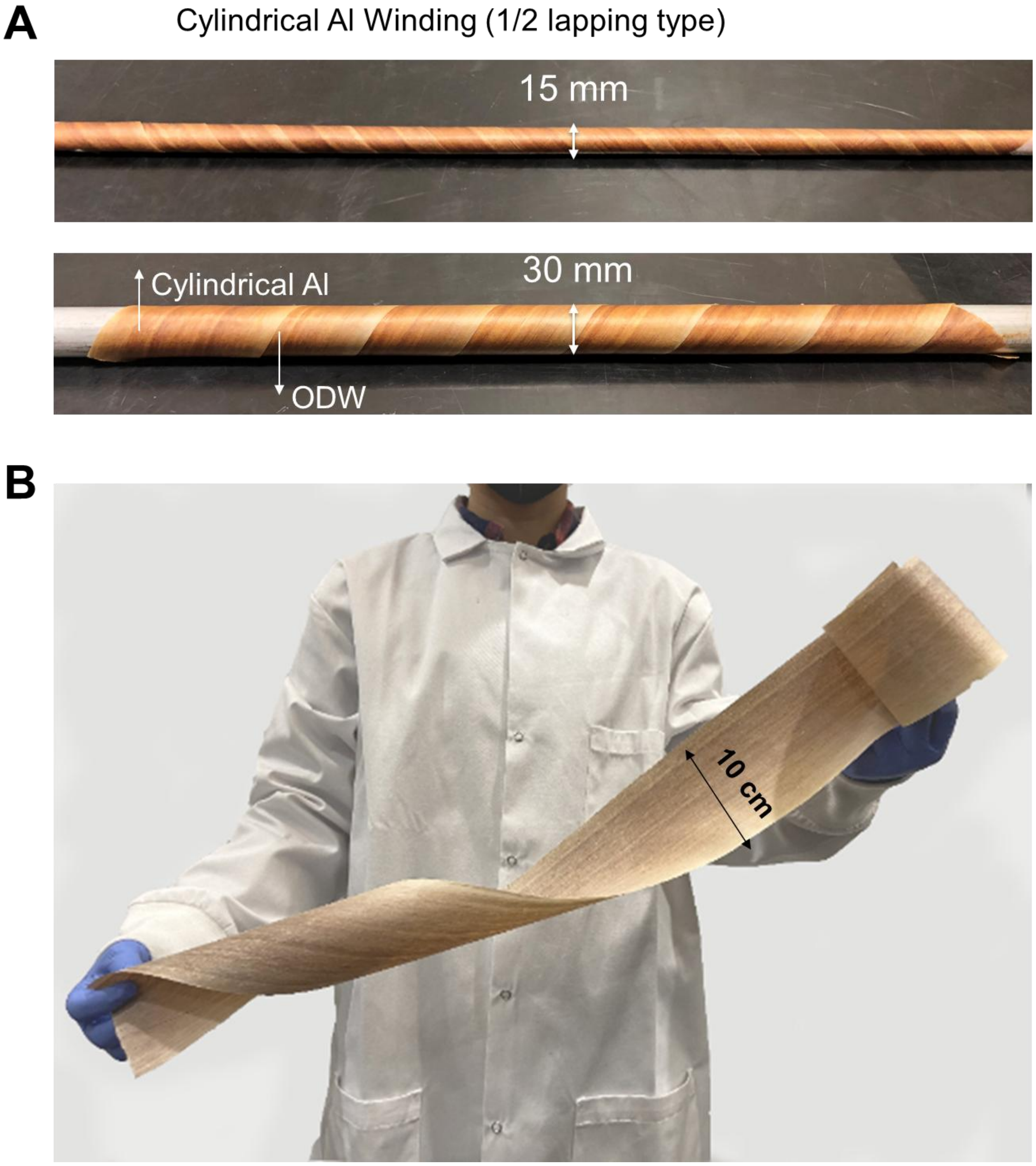

Fig. S10. Demonstration of the winding capability of the ODW. (**A**) Photos of ODW wrapped on cylindrical aluminum rods with a diameter of 15 mm (top) and 30 mm (bottom). The ODW sheets are wrapped with a typical 1/2 lapping method. (**B**) A large-size ODW sample with dimensions of 1.22 meters in length and 0.1 meters in width, showing its flexibility.

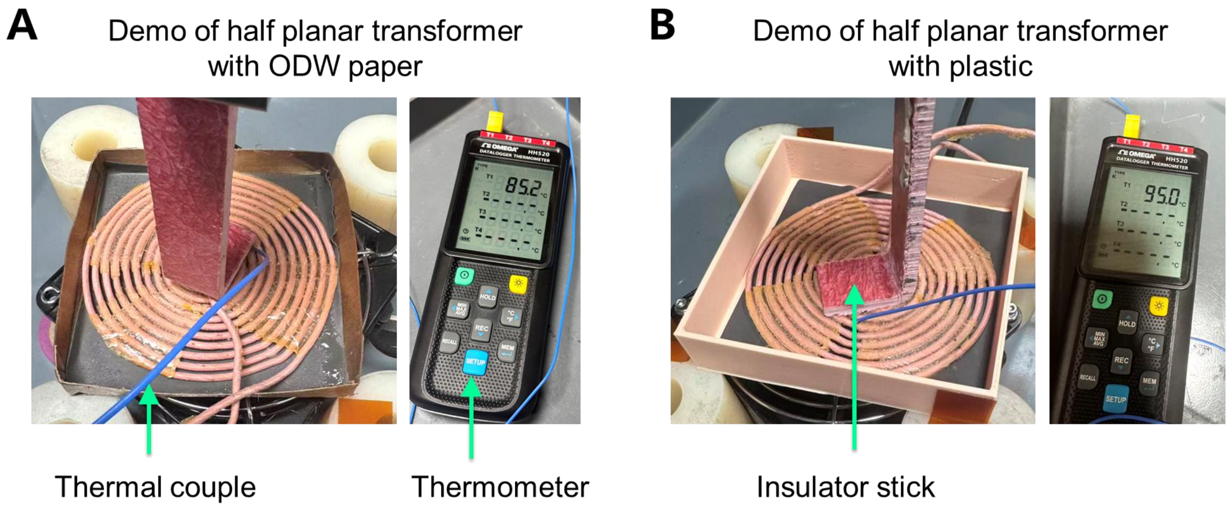

Fig. S11. Temperature measurement of the half planar transformer by using a current of 37 A in the transformer's Litz wire coil to mimic the transformer operation condition. The half planar transformer is made with (**A**) ODW paper and (**B**) plastic, respectively. An insulator stick is applied to provide mechanical pressure between the coil and the thermal couple for accurate hotspot temperature measurement. The temperatures measured by thermometer are similar to the IR images shown in Fig. 5F and 5H. The temperature reduction of ODW displayed by both results is ascribed to ODW's higher thermal conductivity compared to plastic.

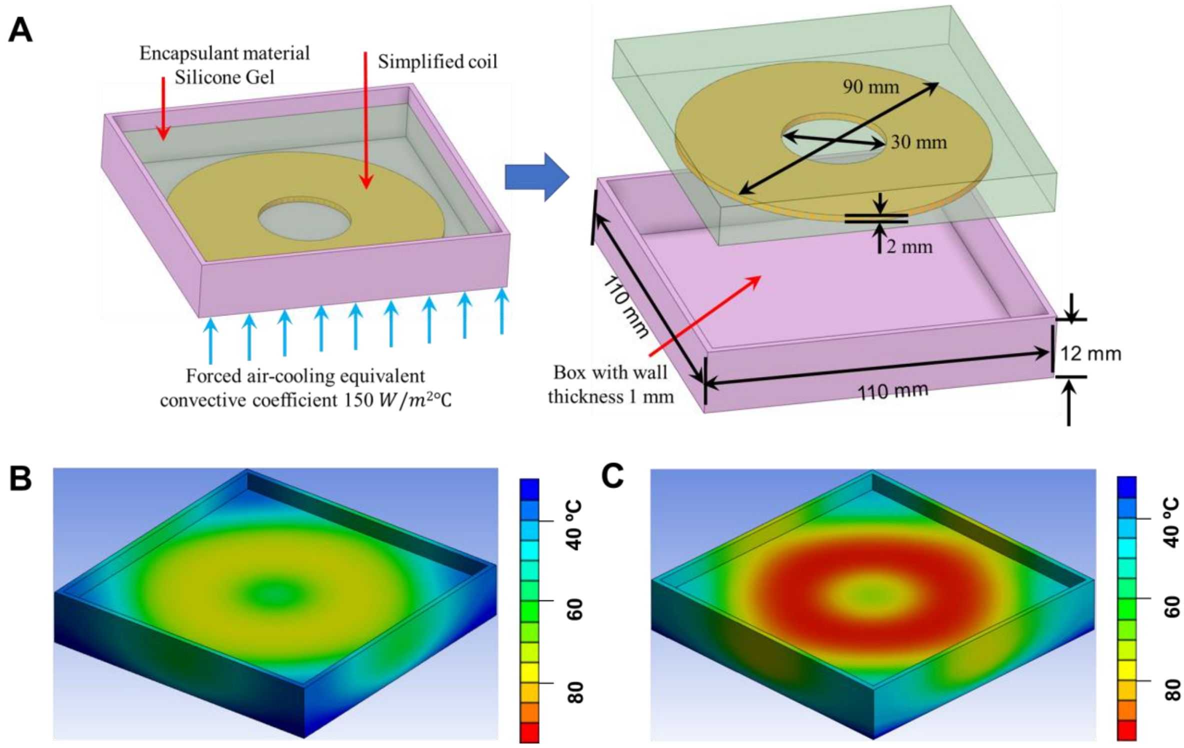

Fig. S12. Model and analysis of the thermal simulation of the planar transformer. (**A**) Schematic diagram of the model dimensions of the insulation box for the thermal simulation. (**B**, **C**) Simulated temperature contour of the planar transformer made of (**B**) ODW and (**C**) plastic.

Table S1. **Properties of each component in oil-impregnated papers, commercial paper, and ODW paper**.

Materials	Dielectric strength (kV mm^{-1})	Relative permittivity	Density (g cm^{-3})	Mechanical strength (MPa)	Thermal conductivity (W m^{-1} K^{-1})
Cellulose	100†	~6.1‡	1.61	N/A	2~14.5§
FR3 ester oil	32.5	~3	0.92	N/A	0.1~0.17
Air	3	1	0.0013	N/A	0.02
Low density OIP	63.3	3.57 ± 0.25	0.71	55.7±15.2	0.12±0.03
High density OIP	71.6	5.47 ± 0.31	0.95	109.6±13.7	0.21±0.01
ODW-0.16 μm	92.6	5.2 ± 0.078	1.3	384.2±13.9	0.33±0.01
ODW-0.56 μm	79.2	---	0.975	---	---
ODW-0.98 μm	69.2	---	0.821	---	---

† All breakdown strength in above table is selected the value at the cumulative failure probability of 63.2%.
‡ All relative permittivity in above table is selected the value at 60 Hz.
§ Thermal conductivity of cellulose is obtained from reference (Nano Lett. 2022, 22, 21, 8406–8412).
